# OSIRISv1.2: A named entity recognition system for sequence variants of genes in biomedical literature

**DOI:** 10.1186/1471-2105-9-84

**Published:** 2008-02-05

**Authors:** Laura I Furlong, Holger Dach, Martin Hofmann-Apitius, Ferran Sanz

**Affiliations:** 1 Research Unit on Biomedical Informatics (GRIB), IMIM, UPF, PRBB, c/Dr. Aiguader 88, E-08003 Barcelona, Spain; 2 Department of Bioinformatics, Fraunhofer Institute for Algorithms and Scientific Computing (SCAI), Sankt Augustin, Germany

## Abstract

**Background:**

Single Nucleotide Polymorphisms, among other type of sequence variants, constitute key elements in genetic epidemiology and pharmacogenomics. While sequence data about genetic variation is found at databases such as dbSNP, clues about the functional and phenotypic consequences of the variations are generally found in biomedical literature. The identification of the relevant documents and the extraction of the information from them are hampered by the large size of literature databases and the lack of widely accepted standard notation for biomedical entities. Thus, automatic systems for the identification of citations of allelic variants of genes in biomedical texts are required.

**Results:**

Our group has previously reported the development of OSIRIS, a system aimed at the retrieval of literature about allelic variants of genes . Here we describe the development of a new version of OSIRIS (OSIRISv1.2, ) which incorporates a new entity recognition module and is built on top of a local mirror of the MEDLINE collection and HgenetInfoDB: a database that collects data on human gene sequence variations. The new entity recognition module is based on a pattern-based search algorithm for the identification of variation terms in the texts and their mapping to dbSNP identifiers. The performance of OSIRISv1.2 was evaluated on a manually annotated corpus, resulting in 99% precision, 82% recall, and an F-score of 0.89. As an example, the application of the system for collecting literature citations for the allelic variants of genes related to the diseases intracranial aneurysm and breast cancer is presented.

**Conclusion:**

OSIRISv1.2 can be used to link literature references to dbSNP database entries with high accuracy, and therefore is suitable for collecting current knowledge on gene sequence variations and supporting the functional annotation of variation databases. The application of OSIRISv1.2 in combination with controlled vocabularies like MeSH provides a way to identify associations of biomedical interest, such as those that relate SNPs with diseases.

## Background

In the last years the focus of biological research has shifted from individual genes and proteins towards the study of entire biological systems. The advent of high-throughput experimentation has led to the generation of large data sets, which is reflected in the constant growth of dedicated repositories such as sequence databases and literature collections. For instance, MEDLINE indexes more than 17 million articles in the biomedical sciences (by April 2007), and it's increasing at a rate of more than 10% each year [1]. In this scenario, literature mining tools are becoming essential for biomedical researchers, as they can be used to extract, manage, integrate and exploit the biological knowledge recorded in the literature. Text mining approaches are being applied to predict the function of novel genes, for the functional annotation of molecules, discovering protein-protein interactions, interpreting microarray experiments, association of genes and phenotypes, to only mention some applications (for review see [1,2]).

The basis of any text mining system is the proper identification of the entities mentioned in the text, also known as Named Entity Recognition (NER). In biology, the entities of interest are genes, proteins, chemical compounds, diseases, tissues, and cellular components, among others. Since naming of biological entities is inconsistent and imprecise, tools that automatically extract the terms that refer to the entities and link them to biological databases are required [3]. NER has been an intense subject of research in the last years, specially for the identification of terms pertaining to genes and proteins [2]. The mapping of a term to a database identifier (also referred to as normalization) is important from a biomedical perspective, because it provides the correct biological context to that term. For instance, mapping a variation entity with a dbSNP [4] identifier allows its unambiguous identification, and in consequence all the information available for this variation can be gathered (organism, genome location, validation status, populations in which the variant has been sequenced, biological sequences where the variant has been mapped, etc.). However, not all NER systems include a normalization step. The first NER tools developed relied on manually devised rules that reflected typical features of gene and protein names as well as contextual information provided by nearby words such as "gene" or "protein" or "receptor" [5,6]. As annotated literature collections (corpora) in which gene and protein names are manually tagged were available, application of machine learning algorithms became feasible [6-10]. Contrasting, dictionary based methods rely on comprehensive lists of terms that are matched against the documents allowing variations in how the terms are written [7,11-17]. The crucial advantage of dictionary based approaches is that they allow the normalization of the entities so far identified.

Contrasting to the extensive research carried out in the field of gene and protein identification, few initiatives have been directed to the task of identification of Single Nucleotide Polymorphisms (SNPs) and other types of sequence variants from the literature. The first approach on this direction was MuteXt, which focused on collecting single point mutations for two protein families: nuclear hormone receptors (NR) and G-protein coupled receptors (GPCR) [18]. MuteXt searched on full text articles looking for protein mutations using regular expressions, achieving a recall of 49.3% and 64.5%, and a precision of 87.9% and 85.8% (for GPCR and NR, respectively). A related approach has been implemented in MEMA [19], in which regular expressions were used to extract variation-gene pairs from MEDLINE abstracts. A difference with MuteXt is that MEMA takes into account variations of the substitution type both at the nucleotide and the amino acid levels. Nevertheless, the MEMA system achieved a higher performance (75% recall and 98% precision) for the identification and extraction of allelic variants from text. The variation tagger Vtag [20] was developed for the identification of several types of sequence variations related to cancer in the literature. It is based on a probability model called Conditional Random Fields. The reported performance of this method is quite good as it reaches 79% recall, 85% precision, and 82% F-score. Although the above mentioned approaches represent nice examples of systems for the identification of variations, some of them are specifically focused on specific protein family classes [18] or diseases [20], and more importantly, none of these methods tackle the problem of the normalization of the variation entities so far identified. These features, however, are incorporated in the OSIRIS information retrieval system (OSIRISv1.1 [21]). OSIRISv1.1 integrates different sources of information and incorporates ad-hoc tools for terminology generation with the aim of literature retrieval about the allelic variants of a gene using the PubMed search engine [22]. Although the use of OSIRISv1.1 helps the researchers in the tedious task of searching literature for sequence variants of genes, there was room for improvement. For instance, the first implementation of OSIRIS uses the PubMed search engine, and does not include a NER system for the identification and tagging of the terms within the text. The present article describes the development of a new version of OSIRIS (herein referred to as OSIRISv1.2). A key aspect was to develop a NER system for identification and normalization of sequence variants of human genes. The new implementation of OSIRIS incorporates its own NER module and is built on top of a local mirror of MEDLINE collection and HgenetInfoDB. HgenetInfoDB is a database that integrates data on human entries from the NCBI Gene database [23] and dbSNP [24]. The NER module is based on a corpus of articles automatically annotated with NCBI Gene identifiers and the new OSIRISv1.2 search algorithm. The new OSIRISv1.2 search algorithm uses a pattern matching based search strategy and a sequence variant nomenclature dictionary for the identification of terms denoting SNPs and other sequence variants and their mapping to dbSNP identifiers. The use of OSIRISv1.2 on MEDLINE abstracts generates a corpus of annotated literature linked to sequence database identifiers (NCBI Gene and dbSNP). The performance of OSIRISv1.2 was evaluated on a manually annotated corpus, resulting in 99% precision, 82% recall, and an F-score of 0.89. As an example, the application of the system for collecting literature for the allelic variants of genes related with two complex diseases (intracranial aneurysm and breast cancer) is presented.

## Results and Discussion

### Development of a NER system for gene sequence variants

Our group has previously reported the development of OSIRISv1.1 [21], a tool for the retrieval of literature associated to the allelic variants of a gene based in the PubMed search engine. With the aim to improve the recognition of variations from text in terms of recall and precision we developed OSIRISv1.2, based on its own NER algorithm and local data sources (MEDLINE abstracts and HgenetInfoDB). In addition, we paid special attention to the normalization of the genetic variants, as it provides biologically relevant contextual information.

**Figure 1:**
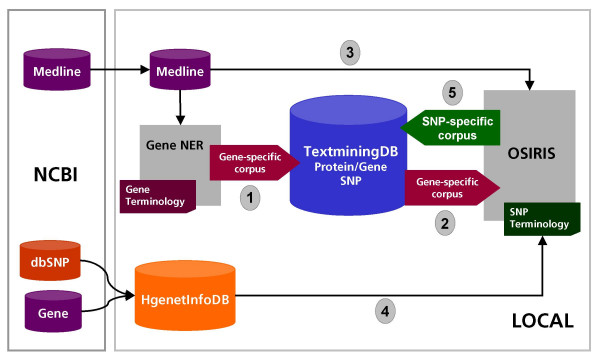
**OSIRISv1.2 system work flow**. The system is based on a local mirror of MEDLINE database and HgenetInfoDB, which integrates information for human Gene and dbSNP databases from the NCBI. The starting point of the system is a gene, for which a set of articles is annotated using the NER tool ProMiner and stored in the TextMiningDB (1). The gene-specific corpus is accessed by OSIRISv1.2 (2) to obtain the MEDLINE citations annotated to a NCBI Gene entry. The corresponding MEDLINE abstracts are retrieved from a local repository (3). In addition, sequence data for each gene and its sequence variants are retrieved from HgenetInfoDB, and this information is used to generate the SNP terminology (4). The next step of OSIRISv1.2 is the search for occurrences of the sequence variant terms in each gene-specific corpus by processing the MEDLINE abstracts. This information (SNP-specific corpus) is returned to the TextMiningDB database (5). The results of OSIRISv1.2, stored in the TextMiningDB, can be accessed through our web interface at [25]. GenDB: data retrieval system used for conversion of the XML files to the files in MEDLINE format, indexing of the MEDLINE files and for their retrieval (internal development of FhG-SCAI by Theo-Heinz Mevissen). ProMiner has been described elsewhere [15]. For simplicity we use in this figure the term SNP to refer to all variations present in the database (SNPs and other types of sequence variants).

The work flow of OSIRISv1.2 is detailed in the Methods section and illustrated in Figure 1. Briefly, OSIRISv1.2 scans MEDLINE abstracts, identifies texts passages corresponding to variation terms, and disambiguates the terms to dbSNP identifiers. The results are stored in the TextMiningDB database, and a web interface for the query and visualization of the results was developed [25]. Results for two diseases used as test cases are currently available in the database. A description of these results can be found in the section "Application of OSIRISv1.2 for finding sequence variants information on a set of aneurysm-related genes". In the following section, a brief description of the user interface is presented.

#### 1. User interface

**Figure 2:**
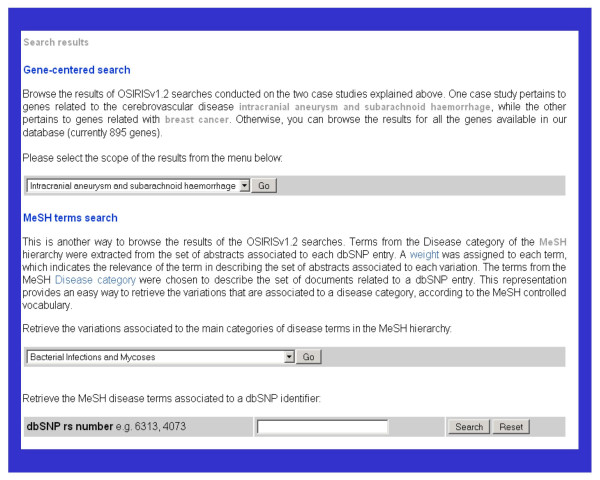
**User interface: search modes**. The user interface offers two possibilities for querying the results of OSIRISv1.2. The results can be queried by a gene-centred search approach, selecting the disease of interest (breast cancer or intracranial aneurysm), or by using MeSH disease terms.

The user interface offers two possibilities for browsing the results of OSIRISv1.2 on the test cases. The results can be queried by a gene-centred search approach on the basis of the related disease (breast cancer or intracranial aneurysm), or by using MeSH disease terms (Figure 2).

##### 1.1. Gene-centred search approach

**Figure 3:**
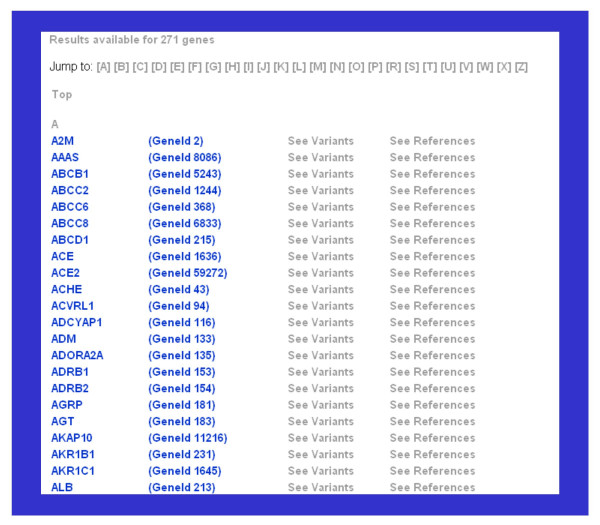
**Gene-centred search results**. By using the gene-centred search approach, a list of the genes for which results were found for the selected disease are presented, sorted in alphabetical order. Two possibilities for browsing the results on these genes are available: "See Variants" and "See References". By clicking the link "See Variants", a page comprising two pieces of information is displayed on the browser: dbSNP-related information for the gene and literature-related information (see Figure 4). By clicking "See References", text passages from all the abstracts found for the sequence variants of the gene are displayed, in a format similar to the one illustrated in Figure 5.

**Figure 4:**
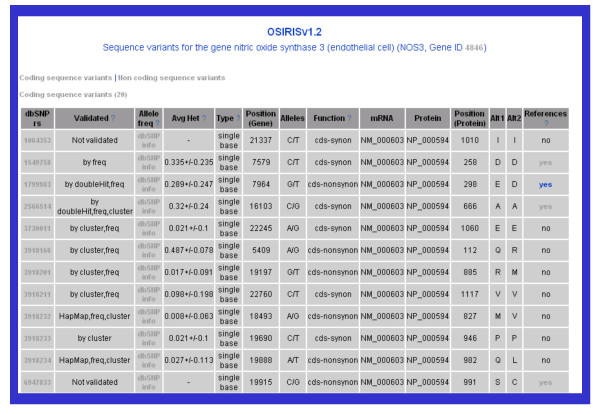
**Information for the sequence variants of the gene**. The link "See Variants", displays a page comprising two pieces of information on the browser: dbSNP-related information for the gene and literature-related information. The first includes the sequence variants described for the gene. The sequence variants are classified according to their mapping to the gene sequence in coding or non-coding regions of the gene. The table includes the following information for each dbSNP entry: the unique identifier from dbSNP (rs number), its validation status and allele frequency, the type of variant (e.g. "single base" for a SNP or "dips" for an insertion/deletion polymorphism), the position of the variant relative to the gene sequence, the alleles, and specifies the mapping or location of the variant (in coding sequences: synonymous or non-synonymous variants; in non-coding sequences: located in introns, splice-sites, untranslated regions, etc.). In addition, the identifiers of the mRNA and protein sequences are provided in the case of SNPs that map to coding sequences. In the case of variations located in coding regions, the position and amino acidic residues of the variation in the protein sequence are shown. The data are provided with hyperlinks to the corresponding data sources (dbSNP and NCBI Gene). The last column of the table indicates if any reference was found for each dbSNP entry, and provides a link to another page where this information is detailed (see Figure 5).

By using this modality of search, a list of the genes for which results were found are presented. Two possibilities for browsing the results on these genes are available: "See Variants" and "See References" (Figure 3). By clicking the link "See Variants", a page comprising two pieces of information is displayed on the browser: dbSNP-related information for the gene and literature-related information (Figure 4). The first includes the sequence variants described for the gene. This information is provided by the database HgenetInfoDB and is organized in tabular format with several hyperlinks. The sequence variants are classified according to their mapping to the gene sequence in coding or non-coding regions of the gene. The table includes the following information for each dbSNP entry: the unique identifier from dbSNP (rs number), the type of variant (e.g. "single base" for a SNP or "dips" for an insertion/deletion polymorphism), the position of the variant relative to the gene sequence, the alleles, and specifies the mapping or location of the variant (in coding sequences: synonymous or non-synonymous variants; in non-coding sequences: located in introns, splice-sites, untranslated regions, etc.). In addition, the identifiers of the mRNA and protein sequences are provided in the case of SNPs that map to coding sequences. In the case of variations located in coding regions, the position and amino acidic residues of the variation in the protein sequence are shown. The data are provided with hyperlinks to the corresponding data sources (dbSNP and NCBI Gene).

**Figure 5:**
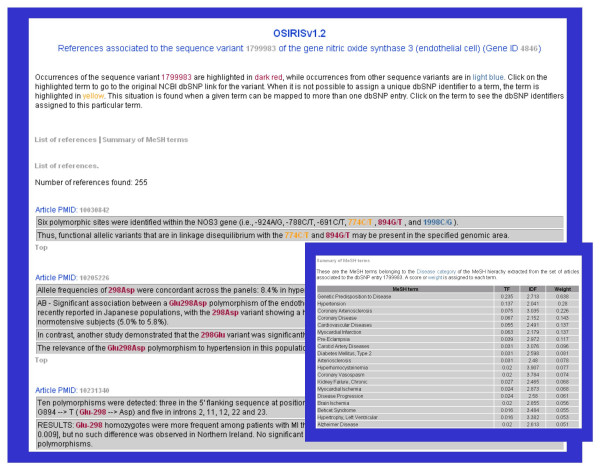
**Literature references for the sequence variants of a gene**. The data is organized by dbSNP entry, displaying the text passages where a term representing the dbSNP entry was identified by OSIRISv1.2. These terms are highlighted to aid visualization and contain hyperlinks to dbSNP. Three colouring codes are used: the terms corresponding to the current dbSNP entry appear in red, in light blue the terms corresponding to other sequence variants found in the text, and in yellow the terms that are mapped to more than one dbSNP identifier. In addition, a table showing the terms from the Disease category of the MeSH hierarchy extracted from the set of abstracts annotated to the variation is provided (inset).

Regarding the literature information, the last column in the table indicates if any reference was found for each dbSNP entry, and provides a link to another page where this information is detailed (Figure 5). In this page, the data is organized by dbSNP entry, displaying the text passages where a term representing the dbSNP entry was identified by OSIRISv1.2. These terms are highlighted to aid visualization and contain hyperlinks to dbSNP. Three colouring codes are used: the terms corresponding to the current dbSNP entry appear in red, in light blue the terms corresponding to other sequence variants found in the text, and in yellow the terms that are mapped to more than one dbSNP identifier. In addition, a table showing the terms from the Disease category of the MeSH hierarchy extracted from the set of abstracts annotated to the variation is provided (Figure 5, inset).

By using this approach, the reader is directly pointed to the text passages where SNPs, for instance, are mentioned. In addition, the PMID of the corresponding article is provided with its hyperlink to the abstract, giving the reader the opportunity to review the complete abstract.

Another view of the results is displayed by clicking "See References", where text passages from all the abstracts found for the sequence variants of the gene are shown. Again, the terms corresponding to the variants are highlighted and contain hyperlinks to dbSNP. Three colouring codes are used: the variants mapped to the current gene appear in red, and in light blue the variants belonging to other genes, in yellow the variants that are mapped to more than one dbSNP identifier.

##### 1.2. MeSH term search approach

**Figure 6:**
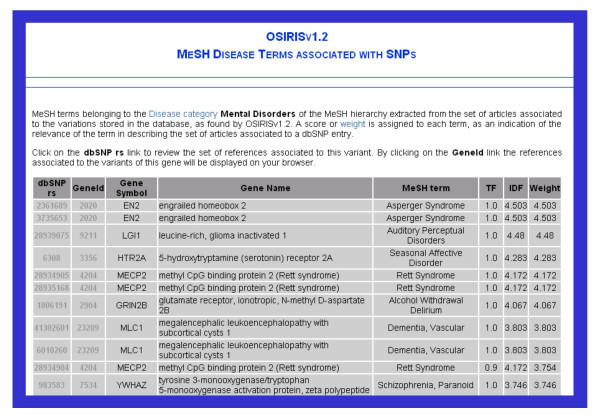
**MeSH terms search results**. As shown in Figure 2, the results can also be queried using the MeSH Disease terms. Variations associated to the main Disease categories from the MeSH hierarchy can be retrieved. The results are organized in a table showing the variants described in association with the disease in the literature, and the weights assigned to each term. This functionality provide a phenotypic description in the disease area of the variations. These results are also shown using the gene-centred search modality.

The results can also be queried using the MeSH Disease terms (Figure 2). Variations associated to the main Disease categories from the MeSH hierarchy can be retrieved. For instance, genetic variants associated with Musculoskeletal Diseases or Cardiovascular Diseases, both examples of broad categories of diseases in the MeSH hierarchy, can be retrieved using this search modality. The results are organized in a table showing the variants described in association with the disease in the literature (Figure 6). Alternatively, the MeSH disease terms associated to an specific dbSNP entry through the literature can be obtained as well. This functionality provides a phenotypic description in the disease area for the allelic variations of a gene. These results are also shown using the gene-centred search modality.

It is worthwhile to mention that not all the terms that refer to variations in the text passages shown in OSIRISv1.2 output are highlighted. This is due to the following reasons: 1 – not all the variants mentioned in the text can be mapped to a dbSNP identifier; 2 – the variant mentioned in the text is mapped to a gene for which a OSIRISv1.2 search has not been performed; 3 – the level of recall of the search algorithm (see below in Evaluation).

In summary, the use of OSIRISv1.2 provides a way to access to the literature reported for the allelic variants of a gene of interest in a few mouse clicks. In addition, it identifies and highlights the terms corresponding to the allelic variants aiding in the visualization of the search results, and therefore saving time for the user.

### Evaluation

An evaluation was carried out to assess the performance of OSIRISv1.2. This kind of evaluation requires a set of reference documents that are judged as relevant or irrelevant for a certain query, i.e. a corpus or gold standard (described in Methods: Evaluation of OSIRISv1.2 performance). A manually annotated corpus was developed for the purpose of the evaluation of the sequence variant extraction and normalization tasks. This is a valuable accomplishment since there is high demand of such standards for evaluation purposes [26]. The corpus is available upon request.

The evaluation consisted in assessing of both the identification of the terms that refer to variations and the normalization of these terms. The result of the evaluation indicated that the algorithm is geared towards high precision (99%) at a recall of 89%, resulting in an F-score of 0.94, for the recognition of variation terms and disambiguation to dbSNP identifiers.

A comparison of the performance of all the current methodologies is shown in Table 1. Both versions of OSIRIS were evaluated on the corpus developed in this study. Regarding the other approaches, although the results of the evaluations carried out in each case are not directly comparable (different corpora and evaluation criteria were used), it serves as a rough comparison with the published methods.

**Table 1:** Comparison of the performance of NER methods for sequence variants.

**Method**	**Precision**	**Recall**	**F-score**
OSIRISv1.1	0.97	0.3	0.46
OSIRISv1.2	0.99	0.82	0.89
Vtag	0.85	0.79	0.82
MuteXt*	0.87	0.57	0.67
MEMA	0.98	0.75	0.85

OSIRISv1.1 and OSIRISv1.2 were evaluated on the corpus described in this study. Performance of other methods correspond to published data for each development. Since the use of different benchmarks and the different evaluation criteria make the results not strictly comparable, the results can only be considered for a rough comparison of the state of the art. *Average of evaluation on two protein families reported

The evaluation for OSIRISv1.2 shows satisfactory results for both precision and recall. The low recall of OSIRISv1.1 could be explained by the inability of the query expansion approach used by the system to recover all the variation specific terms. The recall is increased in the new implementation by improvement of both the recognition of gene and variation terms. Regarding the other approaches, published results indicate that the MEMA system [19] achieved a 75% recall and 98% precision for the recovery of mutations from texts. MEMA uses regular expressions for finding mutations-gene pairs, but it does not provide mapping to dbSNP entries. In this system, the association of a mutation with a gene is done by co-occurrence of both terms in a sentence. Contrasting, OSIRISv1.2 uses co-occurrence and a sequence based approach. The Vtag approach [20], based on a probabilistic model to identify mutation terms, achieves a high recall level, but without providing mapping to sequence database entries. The strategy used by OSIRISv1.2 provides a way to achieve recognition and mapping of variation terms to dbSNP identifiers with high precision and recall, which are very important features for database annotation purposes and mining information from text. In addition, the results of the evaluation indicates that a simple search algorithm performs well in the task of NER and normalization of sequence variants. The recall could be further improved by recognition of complex expressions representing sequence variants, such as "G/A genotype associated with tumour necrosis factor-alpha promoter positions -376 and -244". Future implementations including a broader terminology will probably achieve higher recall levels. Other factor having a negative impact on OSIRISv1.2 recall are the lack of agreement between the numbering of variants position in articles and the one found in databases for some variations.

As mentioned above, the approach used by OSIRISv1.2 to correctly link a variation with a gene is based on co-occurrence of gene and variation terms in the text and sequence mapping. Nevertheless, in some cases it is not possible to assign a variation to a gene in an unambiguous manner. This occurs when two genes and their sequence variants are mentioned in the abstract, and, just by chance, the variants share some of the features required for normalization (the position and the alleles). In such cases, the term will be annotated with two dbSNP identifiers that are mapped to different genes, leading to an ambiguous annotation. These cases are highlighted in yellow in the results displayed on the web.

In order to study in what extent this situation leads to ambiguous annotations, we conducted the following evaluation. We assessed the number of terms referring to a variation that were assigned to more than one dbSNP identifier by OSIRISv1.2. This evaluation was conducted on all the data present in the TextMiningDB (by August 2007). We found that 2.5% (21/841) of the variations had been annotated with more than one identifier from dbSNP. From this set, 0.24% (2/841) resulted from an incorrect identification of a gene term by ProMiner, 1.5% (13/841) were due to multiple annotations in dbSNP, and 0.7% (6/841) resulted from an incorrect assignment of the variation to the term. Multiple annotations in dbSNP occur when a variation has more than one dbSNP identifier (see for example the dbSNP identifiers 1059455 and 41548918 at dbSNP site), and are merged in subsequent updates of the database. This means that 1.5% of the cases are not really ambiguous: the dbSNP identifiers assigned point to the same variation in the gene sequence.

In summary, only 0.7% of annotations are ambiguous due to the inability to distinguish between multiple dbSNP identifiers for a single variation, and lead to an incorrect linkage of variation and gene. The ambiguous annotations are shown in Table 2.

**Table 2:** Detail of ambiguous annotations by OSIRISv1.2

**PMID**	**Term**	**dbSNP identifier**
10087990 10383894 15941956 14557859 15894659 11096344 15668490	142Ser	**3817672** 1799830

14605322	261C > T	**1043428** 11547635

10030842	774C/T	**1549758** 34112109

15372320	79T > C	**40401** 4986964

8655358	Asp86	**17880292** **17885129** 1059350 34095932

17100549	Pro25	**1800471** 2227647

The table show the cases where an ambiguous assignment of a dbSNP identifier to a variation term was obtained. The first column depicts the PMID of the abstracts where the term (second column) was found. The third column shows the dbSNP identifiers assigned to the term by OSIRISv1.2. Highlighted in bold are the correct assignment of dbSNP identifier to that term. In some instances the information from the text does not allow to discern between two SNPs occurring at the same position in the protein sequence but differing only in one of the alternative residues. For example, for the "Asp86" term, both SNPs highlighted in bold represent changes at position 86 of the protein in which an Asp residue is involved. One is a synonymous change while the other is non-synonymous. However, the information extracted from the text is not sufficient to distinguish among these two possibilities (only one allele is described: the Asp residue), and therefore both identifiers are assigned to the term.

### Application of OSIRISv1.2 for finding sequence variant information on a set of aneurysm related genes

Two complex diseases were used as test cases for the system: breast cancer and intracranial aneurysm and subarachnoid haemorrhage. The results can be queried through the web interface [25]. Only results for intracranial aneurysm will be discussed as an example. A summary of the data present in the TextMiningDB database is shown in Table 3.

**Table 3:** Summary of OSIRISv1.2 results available on the TextMiningDB

	**Intracranial aneurysm**	**Breast cancer**	**All**
**PMIDS**	3149	7769	7870
**Gene Id**	271	868	895
**dbSNP Id**	868	2442	2507

Results for the diseases intracranial aneurysm and breast cancer were compiled. The disease-specific corpora were built using PubMed and the queries described in the Methods section. The genes described in each corpora were extracted using ProMiner, and a list of genes specific for each disease was obtained. Then, OSIRISv1.2 searches were performed for each list of genes on the whole MEDLINE collection. Results presented in the table correspond to the MEDLINE collection at August 2007.

Subarachnoid haemorrhage as a consequence of intracranial aneurysm is one of the most devastating cerebrovascular diseases due to its high morbidity and mortality. Cerebral aneurysms are extensions of arterial vessels in the brain. These extensions are balloon-type bulges that form in apparently healthy people with a comparably high frequency (about 2% of the general population have an intracranial aneurysm) [27]. The interaction of genetic as well as environmental risk factors are thought to play an important role in the pathogenesis and prognosis of the disease. In addition to smoking, hypertension, arteriosclerosis and alcohol intake, haemodynamic stress at arterial bifurcations is believed to contribute to the development of aneurysms [28]. Several studies have claimed that the presence of certain allelic variants may increase the individual's susceptibility to develop intracranial aneurysms, and might be used as biomarkers for the risk of aneurysm rupture [28,29]. In this context, the identification of the sequence variants associated to the disease phenotype in specific populations is of high value for early diagnosis and treatment, and also for providing an understanding of the pathogenesis of the disease. OSIRISv1.2 was utilized for collecting sequence variants data for a set of genes related to the disease, and for the identification of SNP terms from MEDLINE abstracts, with the aim of collecting the available information on the variants under study in the disease.

A set of aneurysm-related genes was automatically identified on the basis of their occurrence in abstracts pertaining to the disease. The abstracts related to the disease were retrieved using PubMed search engine with the queries described in Methods and scanned with ProMiner [15]. This resulted in a set of 771 genes, to which 202699 variations are mapped according to dbSNP. Then, OSIRISv1.2 was used to search literature citations for the dbSNP entries mapped to the 771 aneurysm-related genes. Since the searches performed on the abstracts belonging to the aneurysm-related corpora resulted in a small number of OSIRISv1.2 annotations (data not shown), the searches were then conducted in the entire MEDLINE collection. Results were obtained for 271 genes and 868 variants, which were found to be linked to 3149 citations (Table 2). These results represent a collection of the citations that refer to sequence variants on these genes, and are available at [25].

To illustrate the kind of insights that can be obtained using OSIRISv1.2, the gene for the endothelial nitric oxide synthase or eNOS, (NOS3, NCBI Gene Id 4846) will be used as an example. NOS3 is one of the candidates for the pathogenesis of intracranial aneurysm, and its association with the disease has been addressed by several studies [30-33]. Moreover, it might be involved in the observed sex differences in the outcome of subarachnoidal haemorrhage as a consequence of intracranial aneurysms [34]. Endothelial nitric oxide synthase is one of the three isoforms of the enzyme nitric oxide synthase. Nitric oxide (NO) is important in the regulation of vasomotor tone and blood flow by inhibiting smooth muscle contraction and platelet aggregation. Mice lacking NOS3 gene are hypertensive and the combined deletion of NOS3 and apolipoprotein E confers an increased susceptibility to arteriosclerosis. The production of NO is regulated at the level of NOS3 gene expression and activity of the enzyme. The promoter of the gene contains transcription factor binding sites that mediate regulation by shear stress and oestrogens [35].

Several citations were found to refer to variations in the NOS3 gene by OSIRISv1.2. The analysis that follows is based on the literature citations found by OSIRISv1.2 for one of the most studied variants of the gene (dbSNP rs1799983, 255 citations found). Regarding the association with aneurysm, the rs1799983 variant has been described as one of the genetic factors related to the risk of aneurysm rupture in a population from USA (PMID 15796389). Although this finding could not be replicated in a Japanese population (PMID: 17121133), the rs1799983 variant was found to be a mild predisposing factor for abdominal aortic aneurysm, a chronic degenerative condition associated with arteriosclerosis (PMID: 16171581). The putative association of this variant with diseases other than aneurysms has been extensively investigated, as shown by the MeSH disease terms extracted from the set of articles annotated to this variant: Hypertension, Pre-eclampsia, Myocardial Infarction, Alzheimer disease. Deregulation of vascular homeostasis is a common factor among the above mentioned diseases, appearing as a symptom or as a risk factor. The association of the rs1799983 variant with these phenotypes might be reflecting a common mechanism in the disease pathogenesis. The rs1799983 variant, although leads to a conservative change in the protein sequence (Glu to Asp at position 298), would have functional consequences, as suggested by studies showing that the Asp residue is subjected to selective proteolytic cleavage in endothelial cells and vascular tissues (PMID: 10717002, PMID: 11823442) that impairs NO production (PMID: 12065317). However, recent findings have challenged these observations (PMID: 11331296, PMID: 15608562). Thus, it is likely that any relationship between the variant and the disease phenotype is due to this being an indirect marker of the genetic association rather than a direct functional effect. Alternatively, a different functional effect of the variant (e.g. in alternative splicing or in protein folding) might explain the observed clinical features under pathological conditions. Further work is required to clarify the kind of relationship (functional or indirect) of the rs1799983 variant with the disease phenotypes.

## Conclusion

In this report we present OSIRISv1.2, a system for collecting the current knowledge about gene sequence variants from the literature. The evaluation of OSIRISv1.2 on a manually annotated corpus showed that it achieves high precision (99%) and recall (82%) in named entity recognition and disambiguation of terms. The NER system that is the basis of OSIRISv1.2 was combined with a NER system for genes and proteins (ProMiner) that in turn will form the basis of a disease centric information system by integrating NER with disease specific biomedical literature search. This integrated system will allow the extraction of information related to genotype-phenotype relationships in human diseases. The architecture of the system allows incorporation of additional modules, such as NER modules for drugs and diseases, and modules for mining relationships between the entities so far identified. In addition, automatic systems for updating the text mining results allow to keep up to date the extracted information.

To demonstrate the usefulness of the approach, OSIRISv1.2 was applied for the development of a database of literature references associated with allelic variants of genes related with two complex diseases: intracranial aneurysm and subarachnoid haemorrhage, and breast cancer. As illustrated with the example on one of the SNPs of the NOS3 gene, several interesting pieces of information regarding related phenotypes and functional effects of a variation can be easily obtained using OSIRISv1.2. The approach also illustrates the strategy for linking genetic variants of genes present in sequence databases (dbSNP) with disease terms of a controlled vocabulary (MeSH vocabulary) through the literature. Moreover, this approach could be used for developing similar databases focused in other diseases. Although the system has been implemented for human genes, the general approach is easily applicable to other organisms with variation data available (e.g. mouse).

OSIRISv1.2 is focused in identifying the terms from text that represent variations from reference sequence repositories such as dbSNP. The combined use of OSIRISv1.2 (NER and normalization) and any other NER that search for variation entities in general without normalization (any of the methods mentioned in the Background section) would provide with two sets of SNP data that could be of interest to dbSNP curators. In this regard, we anticipate that a suitable application of OSIRISv1.2 would be for supporting the functional annotation of dbSNP entries. This is a relevant issue since the unravelling of the biological consequences of sequence variants remains a challenging task. In addition, elucidation of the functional impact of predisposing SNP alleles identified by genome wide association studies has important implications in terms of understanding the underlying biology of complex diseases and identifying putative therapeutic targets.

## Methods

### OSIRISv1.2 system work flow

A schematic view of the work flow of the system is shown in Figure 1. The starting point of OSIRISv1.2 is a gene, for which a set of annotated abstracts is obtained. The annotation of MEDLINE abstracts with NCBI Gene identifiers is performed with the NER tool ProMiner [15], but any other NER system that performs normalization to NCBI Gene identifiers could be used as well. The ProMiner system was tested in the BioCreAtIvE I and II assessments for the detection of gene and protein names on different organisms [36,37]. The system achieved for all tested organisms excellent results and for human an F-score of 80% with a precision of 83% and a recall of 77%. ProMiner runs performed on the entire MEDLINE collection are used for OSIRISv1.2. This involves a scanning of the abstracts in search for the human entries of NCBI Gene [23] and SwissProt [38] databases. The results of the ProMiner runs are stored in a database (TextMiningDB), and are accessed by OSIRISv1.2 to obtain the corpus of MEDLINE citations annotated to a NCBI Gene entry. The corresponding MEDLINE abstracts are retrieved from a local repository and stored for later processing. In addition, sequence data for each gene and its sequence variants are retrieved from HgenetInfoDB (described below). Using the information obtained from HgenetInfoDB, OSIRISv1.2 generates a dictionary of terms for each variant representing the more frequently used terminology for the variant. The next step of OSIRISv1.2 is the search for occurrences of the sequence variant terms in each gene-specific corpus by processing the MEDLINE abstracts. Once a variation term is found, the dbSNP identifier is assigned to it. This information is returned and back propagated to the TextMiningDB database (Figure 1).

The approach used by OSIRISv1.2 to link a variation with a gene is based on co-occurrence of variation and gene terms in the abstract and sequence mapping. Only the variations mapped to the gene are searched on the text of the gene-specific abstracts (Figure 1). The annotation of variations to a given gene is obtained from dbSNP (for a description see [39]). This procedure allows the identification of the term that refers to a variant and the assignment of a dbSNP identifier to that term (normalization). Normalization of a term provides biological contextual information: for instance, the gene to which this variation is mapped to can be obtained, as well as other gene products (mRNA and protein sequences), and other potentially useful information (population frequency data, state of validation, etc.). In addition, by assignment of a dbSNP identifier to a variation, its linkage to the gene to which the variation is mapped to is established.

### OSIRISV1.2 search algorithm

A search algorithm for identification of sequence variants terms in biomedical text and mapping to dbSNP identifiers was developed. The search is performed for all the variants mapped to the gene of interest on a gene-specific set of abstracts (see Figure 1). The data related to the sequence variants of a gene was obtained from HgenetInfoDB, which is used to build the terminology. The terminology for each variant was generated by a term extension approach according to a set of patterns manually developed after inspecting the literature for collecting usual natural language expressions used to represent sequence variants. Currently, the collection of amino acidic terminology is built by 286 terms, while the nucleotidic terminology is composed of 114 terms for each variant (some examples are depicted in Table 4). The search is performed using pattern matching of the terms from the terminology to text passages. The rationale behind the search algorithm is that for the identification and unique mapping of a text passage representing a sequence variant to a dbSNP identifier, both the position and alleles of the variant are required. Thus, the search algorithm involves two steps: first, the number representing the variant position in the gene, mRNA or protein is searched (for instance in the case of Ala12Val, the number 12 is searched in the text). This step selects sentences that are candidates for having a mention of the variation under search. Then, only the candidate sentences are examined for the complete variation terms. A window of a pre-defined length is extracted from the text around the matched number on the candidate sentence, and the relevant term (e.g. Ala12Val and all its variants: Val12Ala, A12V, ...) is searched by exact-pattern matching in this text fragment. Once a match is found, the position of the pattern in the abstract is computed and stored in the database. This information is then used for visualization purposes, and will be useful for information extraction tasks. As the terms are attached to a dbSNP identifier, once a term is identified in the text, its disambiguation to a database identifier is automatically achieved. The algorithm is implemented in Python 2.4.1.

**Table 4:** Terminology for sequence variants.

**Amino acid terminology**	**Nucleotide terminology**
Q610R	A-161G
R610Q	G-161A
Gln610Arg	G(-161)A
Arg610Gln	-161 A-G
Gln (610) Arg	-161 G-A
Arg (610) Gln	-161 (A-G)
610 Gln > Arg	A(-161)G
610 Arg > Gln	-161 (G-A)

Some examples of the terms generated by the system are shown.

### Databases

A database integrating information on human genes and its sequence variants was developed (HgenetInfoDB). Data for human genes were obtained from NCBI Gene database (release June 2006), and data for sequence variants, including SNPs, indels, etc., were obtained from NCBI dbSNP (build 127). NCBI dbSNP was chosen because is one of the main repositories of variation data and is highly interlinked with other sequence databases within the NCBI. The NCBI Gene database is provided as XML files. For collecting Gene data, the XML file for each human gene was parsed for extraction of relevant information using Python scripts. The data were stored in tab-separated files, suitable for direct importing into database tables. Contrasting to NCBI Gene, dbSNP provides its data as tables that can be directly imported into the database. Only a selection of all the available tables for Homo sapiens (build 126) were downloaded [24] and loaded onto the database (tables SNPContigLocusId, ContigInfo, UniVariation, SNP, SnpFunctionCode and SnpClassCode). Only variants mapped to the reference genome assembly were included in the database. Then, processing and transformation of the data was performed within the database using SQL statements. Among other pieces of information, HgenetInfoDB contains the position of the variant relative to the gene sequence, to the spliced mRNA sequence and to the protein sequence, and information regarding the alternative alleles for each variant. This data is used to generate the terminology. The database was implemented in MySQL version 5.0.15. The current version of the database contains 28998 entries from the Gene database and 4527470 dbSNP entries mapped to a Gene Id.

The results of the OSIRISv1.2 and ProMiner searches were also stored in a relational database (TextMiningDB). This database serves the following purposes: storage of the ProMiner results, which are required for the OSIRISv1.2 searches, storage of the OSIRISv1.2 search results, and for query of results for visualization.

Abstracts in MEDLINE format were used throughout the study. The results here presented correspond to the MEDLINE collection updated to August 16th 2007. The results available on the web server are regularly updated. The abstracts are accessed from a local mirror of MEDLINE repository. The GenDB generic data retrieval system is used for conversion of the XML files to the files in MEDLINE format, indexing of the MEDLINE files and for their retrieval (internal development of FhG-SCAI by Theo-Heinz Mevissen).

### MeSH terms

MeSH [40] terms belonging to the Disease category were chosen to describe the set of articles found to be associated to a dbSNP entry by OSIRISv1.2. The Disease category is formed by 23 sub-categories covering human diseases, with the exception of the C22 branch ('Animal Diseases') which was excluded from the analysis. The branch F03 representing Mental Disorders was added in order to add coverage for psychiatric diseases not included in the above mentioned 23 categories. MeSH terms are assigned to each MEDLINE abstract by experts in the field who read the full text article and choose the most representative terms from the controlled vocabulary for describing the contents of the article. Therefore, MeSH terms represent good descriptors of the topics covered by the article.

Terms from the Disease category of the MeSH [40] hierarchy were extracted from the set of abstracts associated to each dbSNP entry. A weight was assigned to each term, as an indicator of the relevance of the term in describing the set of articles associated to each variation. The weights were computed for the set of documents annotated to a variation by OSIRISv1.2, according to the TF × IDF scoring system, frequently used in text-mining applications [41]. As each MeSH term occurs just once in a document, and we wanted to compute the weight of terms for a set of documents (the set of documents assigned to a variation), the TF was computed as a measure of document frequency of the term in a set of documents. The Term Frequency or TF reflects the importance of the MeSH term in the set of articles associated to a variation (i.e. the fraction of documents with a particular MeSH term), while the Inverse Document Frequency or IDF accounts for the inverse frequency of documents containing the MeSH term in the whole MEDLINE collection. Thus, the Weight or W, defined as TF × IDF, is a composite weight measure for each term in a set of documents. By using this scoring system, a term is assigned a weight that is: highest when the term occurs in most of the documents belonging to the document set; lower when the term occurs in only a small proportion of the document set, or occurs in many documents from the MEDLINE collection; and lowest when the term occurs in almost all the documents from the MEDLINE collection.

This representation, in addition to be useful for describing the topics covered by the set of documents related to a dbSNP entry, provides an easy way to retrieve the variations that are associated to a disease, according to the MeSH controlled vocabulary. A search functionality using the MeSH disease terms was also included in the web interface (see description in the Results and Discussion section: User interface).

### Evaluation of OSIRISv1.2 performance

Our goal was to assess the processes of entity recognition and subsequent normalization of genetic variations. The entity recognition of gene and protein terms by ProMiner has been evaluated elsewhere [36,37]. The performance of OSIRISv1.2 was estimated by computing its precision (number of correctly identified entities divided by the total number of entities identified), recall (number of correctly identified entities divided by the total number of entities in the text) and F-score (weighted average of precision and recall, (2·precision·recall)/(precision+recall)). The values of precision and recall were computed for the combined process of entity recognition and normalization: that means that a match was considered a true positive when a genetic variation term was identified and disambiguated to the correct dbSNP identifier; otherwise, it was computed as false positive. Thus, the assignment of a correct dbSNP identifier to an entity found in the abstract text implies the establishment of a correct linkage between the gene and the variation.

Some of the corpora developed for evaluation of text mining applications in the biomedical domain [26] were considered with respect to their suitability for the evaluation of OSIRISv1.2. They were GENIA corpus [42], PennBioIE oncology and PennBioIE cyp [43]. However, none of them were found suitable for the task because of the low occurrence of sequence variant terms in the documents included in these corpora. Another source of sequence variant annotation of the literature is provided by the linkage between dbSNP and PubMed supplied by the NCBI. These links were studied to assess if they could be used for compiling a set of articles annotated with dbSNP entries. It was observed that the aforementioned links were only established between articles that describe genotyping projects involving large fragments of chromosomes and the dbSNP entries that are mapped to these chromosomal fragments. These links are generated by connecting submissions to dbSNP to PubMed records of publications cited at the time of submission [44]. For instance, for 76,438 dbSNP entries only 32 different articles were annotated. Moreover, only a small proportion of the abstracts included in their text a direct mention of a specific dbSNP entry. In the case of the aforementioned 32 articles, only 10 abstracts mentioned specific SNPs. In consequence, the use of the dbSNP and PubMed links for OSIRISv1.2 assessment was discarded.

Thus, a *de novo *corpus was developed for the purpose of OSIRISv1.2 evaluation. A starting set of 578 abstracts was obtained using the PubMed [22] search engine and the following search query:

"Pathological Conditions, Signs and Symptoms" [MeSH] AND "Polymorphism, Single Nucleotide" [MeSH] AND "Humans" [MeSH] AND hasabstract [text] AND English [lang] AND ("2004/01/01" [PDAT] : "2005/01/01" [PDAT]) AND "Chemicals and Drugs Category" [MeSH]

This set is focused to a field of interest to our group and limited to years 2004 and 2005. As a first step in the annotation process, the set of abstracts was carefully reviewed to determine if it contained explicit mentions of sequence variants in the text that allow their mapping to a dbSNP identifier: the position of the variation in the sequence, one or both alleles, and the gene to which the variation is mapped to. Only 311 abstracts included this information and were retained in the corpus. Thus, these abstracts contained mentions of variations that could potentially be normalized to dbSNP identifiers (although not all of them were disambiguated to a dbSNP identifier, see below). The next step consisted in the manual annotation of gene and sequence variant occurrences with their database identifiers. Since the process of manual annotation is very laborious and time consuming, a subset consisting of the first 105 abstracts (in terms of date of publication) was selected to perform the full annotation. The quality of the annotation was prioritised in front of the size of the corpus. Nevertheless, the size of the corpus (n = 105) is similar to other corpora used in other evaluations carried out in the field [19].

The annotation of the abstracts consisted in manual identification of mentions of genes and variations and their disambiguation to database identifiers. The NCBI Gene database was used in the case of genes and HgenetInfoDB in the case of variations. The text inspection was performed at the level of title and abstract and the resulting annotations were recorded in the corpus as an XML file using the Vex editor [45] in the framework of the Eclipse platform [46]. The result of the annotation is summarized as follows: from the set of 105 abstracts, 102 abstracts contained gene mentions that were disambiguated to a NCBI Gene identifier. The remaining 3 abstracts contained gene mentions that could not be normalized. Fifty five abstracts contained mentions of variations normalized to dbSNP entries (109 variations), while 50 abstracts contained mentions of sequence variants that could not be mapped to dbSNP identifiers (155 variations). The corpus is available to other researchers upon request.

### PubMed search queries

The search queries used to select the set of articles related to the diseases of interest are detailed below. Results presented in this article correspond to searches conducted on MEDLINE database updated to August 2007.

For intracranial aneurysm and subarachnoid haemorrhage:

("intracranial aneurysm" [TIAB] NOT MEDLINE [SB]) OR "intracranial aneurysm" [MeSH Terms] OR cerebral aneurysm [Text Word]

("subarachnoid hemorrhage" [TIAB] NOT MEDLINE [SB]) OR " subarachnoid hemorrhage" [MeSH Terms] OR subarachnoid hemorrhage [Text Word]

For breast cancer:

("breast neoplasms" [TIAB] NOT Medline [SB]) OR "breast neoplasms" [MeSH Terms] OR Breast Cancer [Text Word]

The search query used for compiling the corpus was:

"Pathological Conditions, Signs and Symptoms" [MeSH] AND "Polymorphism, Single Nucleotide" [MeSH] AND "Humans" [MeSH] AND hasabstract [text] AND English [lang] AND ("2004/01/01" [PDAT] : "2005/01/01" [PDAT]) AND "Chemicals and Drugs Category" [MeSH]

## Authors' contributions

LIF participated in the design of the system, implementation, evaluation and analysis of the results and prepared the manuscript document. HD and MHA contributed to the design of the system, and HD contributed to the database design and implementation. FS participated in the design and coordination of the work. All authors contributed to the manuscript, and read and approved its final version.
